# *In vivo* induction of male sexual behavior in zebrafish by adding agents to water

**DOI:** 10.1371/journal.pone.0300759

**Published:** 2024-08-01

**Authors:** Saokat Ahamed, Mohammad Maksudul Hassan, Umme Habiba Mustary, Mohammad Tohidul Amin, Toshinobu Tokumoto

**Affiliations:** Department of Bioscience, Graduate School of Science and Technology, National University Corporation Shizuoka University, Shizuoka, Japan; University of Hyderabad, INDIA

## Abstract

Successful fertilization in fish mating occurs when egg maturation in the ovary of the female, ovulation, sperm maturation in the testis of the male, and reproductive behaviors in both sexes are triggered in synchrony. The male sexual behavior of fish is induced by hormones and pheromones. In a previous study, we demonstrated that externally applied hormones added to the water can induce oocyte maturation and ovulation in female zebrafish. Here, we attempted to establish a similar method to induce the sexual behavior of male zebrafish. The male sex steroid testosterone (Tes) triggered sexual behavior within several hours *in vivo* when administered directly into the surrounding water. A selective agonist for membrane progesterone receptor (mPR), Org OD-02 (Org), also induced sexual behavior. Through trials of various combinations of compounds, we found that the most effective conditions were achieved by treatment with a mixture of testosterone (Tes) and Org. The effect of treatment was evaluated by the number of fertilized eggs obtained by pairing with females with induced ovulation *in vivo*. The period necessary for the induction of male sexual behavior was evaluated by time course experiments. The success rate of mating and the number of fertilized eggs reached the maximum level at 3–4 hours of treatment. The duration of hormonal treatment was confirmed by counting the number of hooking occurrences, which is the final cue to induce spawning by females. In summary, we have established a method to induce male sexual behavior in zebrafish *in vivo*. The method can be used to obtain fertilized eggs in zebrafish by simply adding agents into the water.

## Introduction

The induction of reproductive behavior in fish is caused by a relatively long period of gonadal preparation by hormones and a short period of behavioral induction by pheromones.

The manifestation of sex-specific behaviors in fishes is determined at an early stage through the influence of steroids [1]. In adult teleost fishes, the ability to exhibit either male or female behaviors can be observed with exposure to suitable hormones and pheromones within the appropriate environmental conditions [2].

Research on the elicitation mechanisms of reproductive behavior in fish commenced with the goldfish species, and a fundamental narrative was built subsequent to the identification of pheromones [3]. The prevailing hypothesis posits that the regulation of reproductive behavior, specifically pertaining to sex-specific traits, is primarily governed by hormonal control, with a particular emphasis on steroid hormones. Pheromones, in turn, are believed to function as the ultimate trigger in this intricate system. Hormones function as pheromones when they are excreted from the fish’s body, thus giving rise to the phrase "hormonal pheromone" [2]. Subsequent comprehensive investigations into the processes underlying the actions of hormones and pheromones in diverse fish species have revealed that the mechanisms governing sexual behavior exhibit divergence, with distinct species-specific variations [4].

Zebrafish are a model organism for fishes. In light of the advancements in genome editing technologies, there has emerged a necessity to devise methodologies that can artificially stimulate spawning in females and induce reproductive behavior in males. This is crucial for the preservation of gene-disrupted strains, particularly in cases when individuals exhibit diminished reproductive capabilities.

It is noteworthy that the level of membrane progesterone receptors (mPRs) expressed in the olfactory mucosa (OM) of zebrafish is significantly high [5]. According to genomic study, the mPR with the highest expression level was identified as mPRγ, also known as paqr5b. It is plausible that mPRs may serve as receptors for the hormonal pheromone 17α, 20β-dihydroxy-4-pregnen-3-one (17α, 20β-DHP) or its sulfate form, 17,20β-DHP-S. The process of screening involved the utilization of recombinant mPRs that were expressed in cultured cells. This screening approach successfully discovered a specific agonist, known as Org OD-02 (Org), which selectively activates the mPRs [6]. Org showed agonistic activity in zebrafish oocyte maturation and ovulation [7].

In a previous study, it was demonstrated that the administration of endogenous steroid hormones topically could effectively stimulate the process of oocyte maturation and subsequent ovulation in live zebrafish specimens [8]. Adding 17,20β-DHP to the water induced zebrafish spawning. This *in vivo* induction method is applicable as a new fish reproduction method to obtain ovulated eggs for fertilization. Similar to 17,20β-DHP, Org showed the same activity in *in vivo* induction in zebrafish.

In this study, we tried to establish conditions for inducing male sexual behavior in the same way as female sexual behavior in zebrafish. By various trials, we identified the optimal conditions to induce male sexual behavior. Interestingly, the agonist for mPRs, Org, showed inducing activity for zebrafish male sexual behavior. By combining the induction of sexual behavior and spawning through hormone treatment of males and females, respectively, we established a method to artificially obtain fertilized eggs of zebrafish by the simple method of adding hormones to water.

## Results

### Externally applied hormones induce male sexual behavior *in vivo*

**Fig 1:**
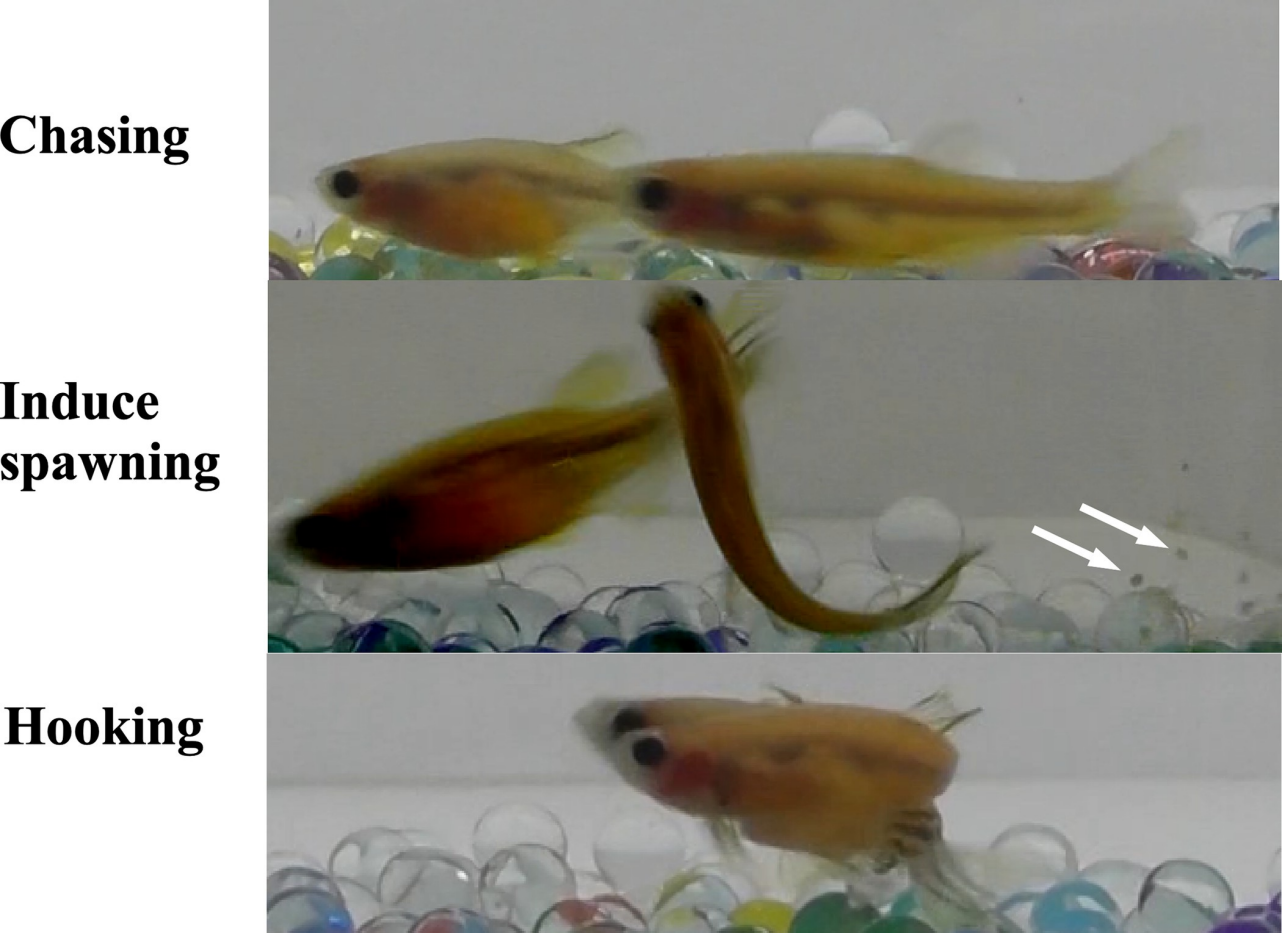
Compounds added to water induce male zebrafish sexual behavior. Photographs of chasing behavior, spawn-inducing behavior and hooking behavior of males artificially induced by adding agents to water.

**Fig 2:**
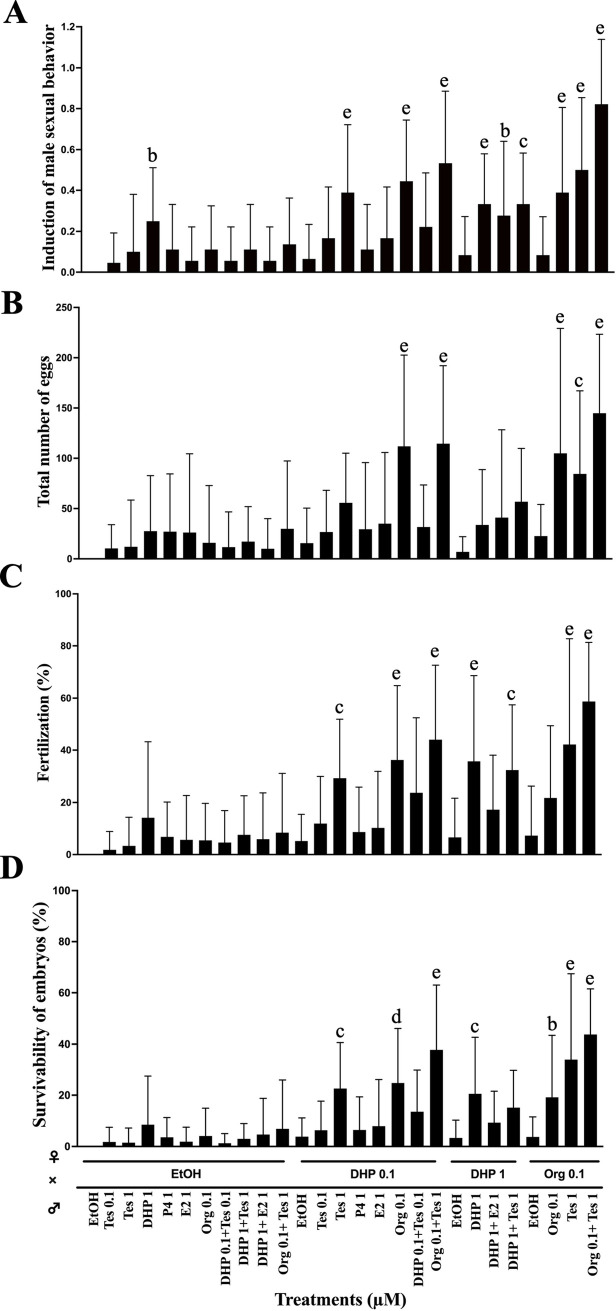
Compounds added to water induce male zebrafish sexual behavior. Male zebrafish were treated with various combinations of compounds for four hours. Stimulation levels of sexual behavior were evaluated by pairing with females with ovulated eggs inside the body. The concentrations of compounds are indicated under each column. Each compound was added to water at a 10,000-fold dilution from a stock solution in ethanol. Male sexual behavior was observed for 20 min and classified as only chasing or chasing and inducing spawning. (A) The behavior of only chasing was scored as 0.5, and chasing and inducing spawning was scored as 1.0. (B) The total egg number obtained by mating was compared among treatments. (C) Survival rate of fertilized eggs obtained by mating. Each value represents the mean of data from three or more trials with three different males (n≧9). Vertical lines indicate standard deviation. One-way ANOVA was used to assess significant differences among the treated groups. Different letters represent significant differences among the data. b, P value < 0.05, c, P value < 0.01, d, P value < 0.001, e, P value < 0.0001. The mark ‘a’, which indicates that the groups did not show significant differences from the control (ethanol, EtOH), is omitted. All the low data represented in the figure is available in S1 Table, also data of statistical analysis is available in S2 Table.

To evaluate the effect of hormones on zebrafish male sexual behavior, we administered various hormones directly into the water in which we held live zebrafish. As an initial trial, we treated male zebrafish for 4 hours with an *in vivo* treatment used to induce ovulation in females. After treatment, male zebrafish were paired with ovulation-induced females. When male fish sexual behavior was induced, male fish chased females from behind and induced female spawning, and fertilized eggs were obtained. However, at least two stages of male tracking behavior were distinguished: one in which the male only chased the female (Fig 1) (representative behavior shown in S1 Movie), and the other in which the male chased the female and could lead her to spawn (Fig 1) (S2 Movie). Therefore, we quantified the activation state of sexual behavior by assigning a value of 0.5 for the intermediate stage of sexual behavior in which the animals only chased and 1.0 for the highest level of activation in which they chased and could be induced to spawn. We conducted trials of the induction of male zebrafish sexual behavior by various hormones and related compounds at different concentrations. As expected, the external application of some hormones induced male sexual behavior *in vivo*. Fig 2 summarizes the results. The solvent for the hormones, ethanol, did not show any effect, but testosterone (Tes) and Org showed inducing activity. Maximum effects on the induction of male sexual behavior were observed when a mixture of Tes and Org was added to the water. The number of fertilized eggs obtained and the fertilization rate were examined to determine whether male sexual behavior was induced or induced to release sperm. As shown in Fig 2, the number of fertilized eggs and the fertilization rate showed a trend consistent with the induction rate of male sexual behavior. These results indicate that *in vivo* hormone treatment through water for 4 hours can induce full sexual behavior with sperm release in zebrafish.

**Fig 3:**
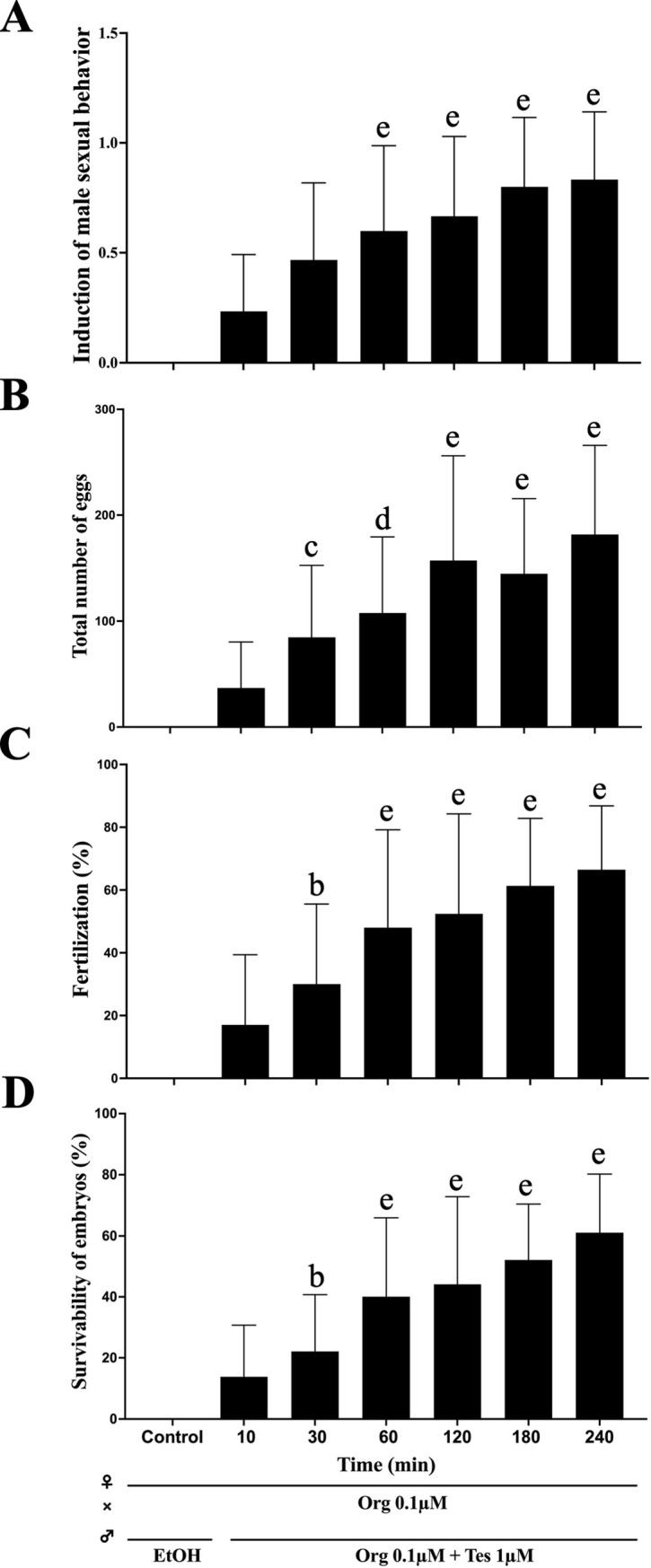
Effect of the length of treatment during induction of male sexual behavior *in vivo*. Male zebrafish were treated for different periods as indicated. After treatment, male zebrafish were paired with females with induced ovulation. (A) The behavior of only chasing was scored as 0.5, and chasing and inducing spawning was scored as 1.0. (B) The total egg number obtained by mating was compared among treatments. (C) Survival rate of fertilized eggs obtained by mating. Each value represents the mean of data from three or more trials with three different males (n≧9). Vertical lines indicate standard deviation. One-way ANOVA was used to assess significant differences among the treated groups. Different letters represent significant differences among the data. b, P value < 0.05, c, P value < 0.01, d, P value < 0.001, e, P value < 0.0001. The mark ‘a’, which indicates that the group did not show significant differences from the control (ethanol, EtOH), is omitted. All the low data represented in the figure is available in S1 Table, also data of statistical analysis is available in S2 Table.

**Fig 4:**
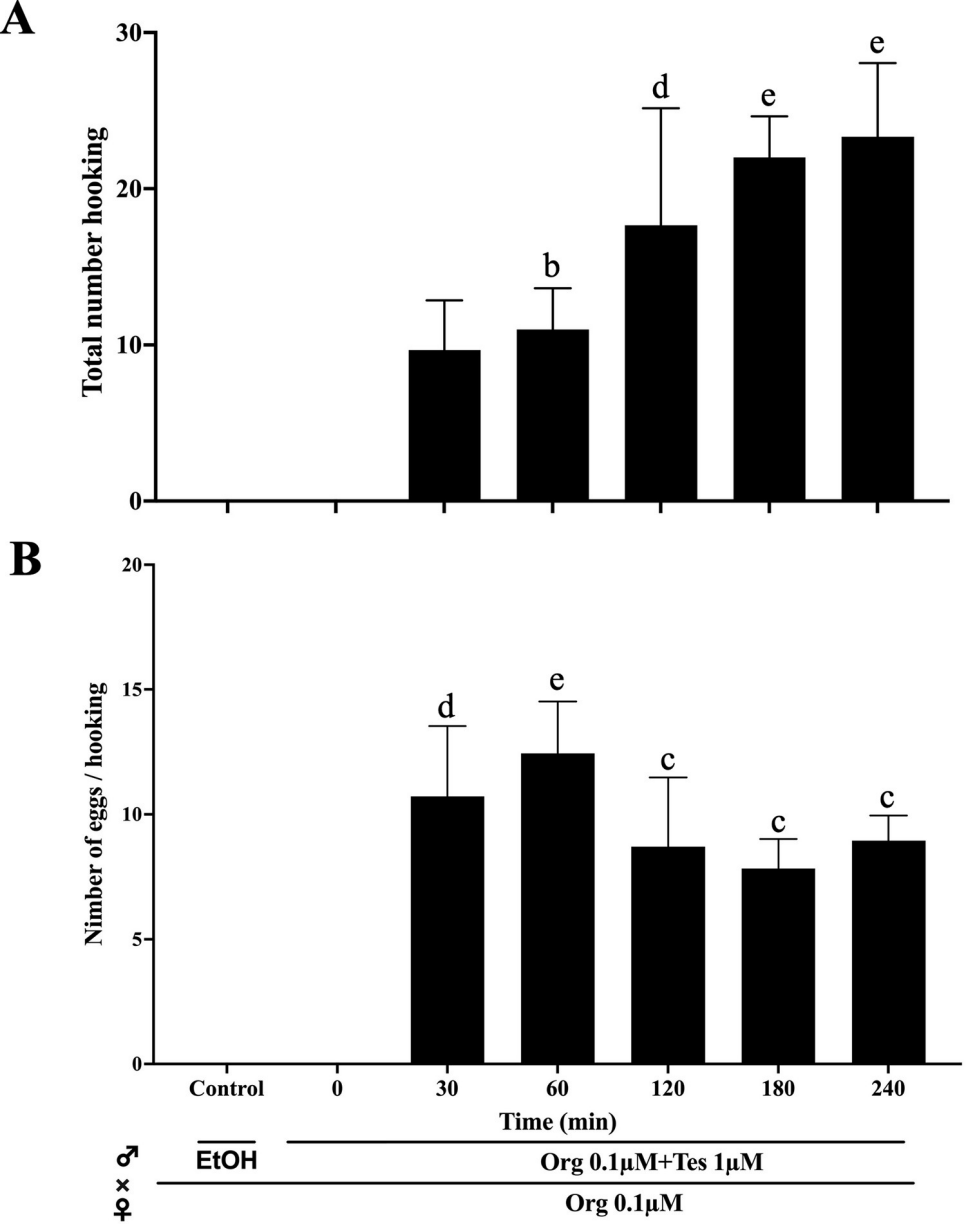
Effect of the length of treatment during induction of hooking behavior *in vivo*. **(**A) The total number of hooking events during 20 min of mating was counted by observation of superslow movies. (B) Spawned egg numbers per hooking event in each treated group are indicated. Each value represents the mean of the data from three trials with three different males (n = 3). Vertical lines indicate standard deviation. One-way ANOVA was used to assess significant differences among the treated groups. Different letters represent significant differences among the data. b, P value < 0.05, c, P value < 0.01, d, P value < 0.001, e, P value < 0.0001. The mark ‘a’, which indicates that the group did not show significant differences from the control (ethanol, EtOH), is omitted. All the low data represented in the figure is available in S1 Table, also data of statistical analysis is available in S2 Table.

Next, we examined how long it took to induce sexual behavior. This allowed us to estimate whether the externally induced effects were hormonal or pheromone effects. The effects of the mixture of Tes and Org, which showed the greatest effect, were examined at different treatment times (10 and 30 minutes and 1, 2, 3, and 4 hours). The results showed that both the effects of induction of reproductive behavior and the number of fertilized eggs increased with time, reaching a maximum at 3–4 hours (Fig 3). However, there were a number of cases where induction occurred even after 10 minutes of treatment. High-speed imaging analysis revealed that the final stage of male zebrafish sexual behavior is the hooking of the female’s body with the dorsal fin to induce spawning [9] (Fig 1) (S3 Movie). Therefore, we also examined the effect of hormones on the induction of male sexual behavior by counting the number of hooking events induced *in vivo* (Fig 4). The frequency of hooking increased with the duration of hormone treatment, reaching a maximum at 3–4 hours, a similar trend to the number of eggs obtained. From the number of spawned eggs and the number of hooking events, the number of eggs spawned per hooking event was calculated (Fig 4B). The overall average number of eggs spawned per hooking event was 9.7 ± 1.8 eggs/hooking. Although the total number of spawned eggs was increase 2–4 hours (Fig 3B), number of eggs/hooking at 1 hour is significantly higher than others. The reason for this result is due to the limited number of eggs laid by the females. Although the males were more active and hooking frequency was higher in the 3 and 4 hours treatments (Fig 4A), the number of eggs per hooking was extremely low in the latter half of the observation period (20 min). Therefore, the number of eggs per hooking is relatively low in this calculation. Also, the result suggested the possibility of degradation of Tes and Org during incubation. Therefore, we measured the half-life of Tes and Org by HPLC analysis (S1 Fig). The result showed that the half-life of Tes and Org in our experimental condition was significantly longer (Tes: 50.4 hours, Org: 74.5 hours) than the incubation periods. It can be concluded that the result of maximum number of eggs obtained at 1 hour treatment was not due to the degradation of Tes and Org.

**Fig 5:**
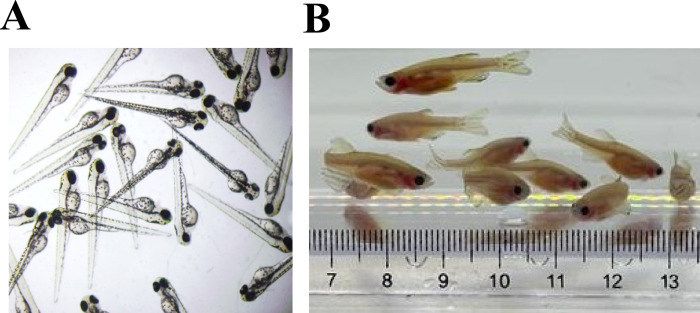
Juveniles produced by artificial mating with mPR agonists developed normally. Photographs of embryos (A) and adult fish (B) from the pairing of activated males and females with induced ovulation by Org.

As shown in a previous report, Org induces ovulation identical to physiological ovulation. The method can be used as a new way for artificially inducing ovulation in zebrafish. As described in previous, *in vivo* hormone treatment with Org can induce ovulation in female as same as DHP [7]. We calculated induction rate of female in some trials in this study. The result was the same as previous report, 86.7% at 0.1 μM of Org (n = 13/15). In this study, we showed that Org also induces male sexual behavior. Combining the methods by adding hormones to the water can induce male as well as female sexual behavior. Juveniles produced by combined treatment with Org developed normally (Fig 5). It is now possible to harvest zebrafish through a simple method of adding agents into the water.

## Discussion

In this study, we aimed to induce male zebrafish sexual behavior by adding a solution of agents into the water as previously described in females. The results showed that Tes and Org successfully induced sexual behavior in fish kept in water containing these agents. Sexual behavior was induced *in vivo* by this method and took several hours (3–4 hours) to reach maximum levels. Since the time taken to induce male sexual behavior was not significantly different from that for oocyte maturation and ovulation in females, it can be concluded that steroids penetrate the fish body and accumulate in the testis. Our tracer experiments on the uptake and concentration of hormones in African clawed frogs (*Xenopus laevis)* have shown that steroid hormones are concentrated in the ovaries and testes over a period of several hours [10]. The *in vivo* induction method to induce spawning was also effective in *Xenopus laevis* [11]. The addition of a mixture of progesterone and estradiol to the breeding water induced spawning in females. Furthermore, testosterone treatment of males induced sexual behavior in male frogs [10]. Finally, we established a simple method to obtain fertilized eggs without hormone injections by adding hormones to the water of females and males in *Xenopus laevis*. Similarly, we performed tracer experiments in zebrafish. As in frog, a 5.4-fold higher concentration of Tes was found in testis than in muscle (S3 Table). There should be some mechanisms by which steroids are transferred to the reproductive organs. Extremely high concentration was detected in gills and digestive tissues. The results suggested that Tes was absorbed through the gills and digestive tissues and transferred to the testes. We also tested the relationship between Tes incorporation rate and fish body weight by tracer experiments (S4 Table). The incorporation rate increased with fish body weight. It could be speculated that the amount of incorporation of compounds increased with the size of the tissues for incorporation (gills and digestive tissues).

The effect can be elicited as quickly as 10 minutes, which seems to suggest that it has a pheromone effect, but it takes 3–4 hours to show maximum effect. We believe that the animals that were induced by the short treatment were already in a physiological state of sperm readiness in the testes and were ready to release sperm. It is thought that the differences in physiological readiness among individuals indicated an increase in reproductive behavior along the time axis.

Interestingly, the mPR selective agonist Org exhibited a potent inducing effect on male sexual behavior *in vivo* as same as ovulation in female. Thus, the mPR signaling pathway is believed to be involved in the induction of male sexual behavior by affecting the nongenomic actions of steroids. While a number of reports have described the induction of sexual behavior by hormones and pheromones, only a small proportion have investigated induction by nongenomic actions.

Although mPR has been shown to be expressed in sperm and involved in the activation of sperm motility [12], the target of *in vivo* treatment is thought to be involved in the induction of final sperm maturation or the release of sperm from the testis rather than in the sperm itself since it takes several hours before the treatment effects are seen. mPR-mediated nongenomic actions, such as the induction of oocyte maturation, are expected to be involved in the induction of final sperm maturation or the release of sperm. One of the mPR subtypes, mPRγ (paqr5b), was found to be highly expressed in the olfactory mucosa (OM), an organ for smelling that detects pheromones and hormones [5]. Org may have acted on mPRγ in the OM as a pheromone, and its stimulation may have caused testicular activation via the cranial nerves. Although it is possible that Tes also acts secondarily via the cranial nerves, it is reasonable to assume that it acts directly on the testes, similar to the action of progesterone on the ovaries.

In this study, we established a new method to induce reproductive behavior in male zebrafish without hormone injections by adding a hormone solution to the water. By combining this method with the previously described method of inducing female ovulation, a new artificial egg collection method was established. This method can be used for strain maintenance of zebrafish and is a remarkable method that has the potential to be applied as an artificial egg collection method for other fish species.

## Methods

### Ethics statement

All zebrafish experiments were carried out with approval from the Institutional Ethics Committee of Shizuoka University, Japan (approval no. 2022F-3 and 2023F-9); the guidelines set by this committee for the usage of animals were strictly followed. The study was carried out in compliance with the ARRIVE guidelines [13].

### Materials

Zebrafish were raised and kept under standard laboratory conditions. Fish used for experiments were maintained in an outflow culture system maintained at 28.5°C on a 14 h light/10 h dark cycle [9]. Steroid hormones (progesterone, testosterone, 17β-estradiol) and diethylstilbestrol (DES) were purchased from Sigma Chemical Co. (St. Louis, MO). 17,20β-DHP was purchased from Toronto Research Chemicals (Toronto, Canada). Org OD-02 was obtained from AXON MedChem BV (Groningen, Netherlands). Other chemicals were purchased from Wako Pure Chemical Industries, Ltd. (Osaka, Japan).

### *In vivo* induction of male sexual behavior

Male zebrafish showing no sexual behavior (avoid males naturally showing behavior before experiments) were selected from a mixed group of 10–50 males and females held in a 20 cm x 25 cm square acryl case that was 25 cm high and fed with continuous outflow water in the morning. After turning on the light in the morning, some male fish showed chasing behavior against females. These males were removed from the experimental fishes. Males were transferred into a glass case containing 100 ml of water per fish. Fish were exposed to agents *in vivo* by adding each agent into the water (from a 10,000-fold stock in ethanol) at 28.5°C. After incubation, treated male zebrafish were transferred into a pairing case with ovulation-induced females. The behavior of fishes in the pairing tank was captured by a video camera for 20 min (SZX12, Olympus, Japan). After 1 hour of pairing, spawned eggs were collected, and the number of eggs was counted. The fertilization rate of eggs was also evaluated by observing whether cleavage of cells occurred.

### Counting of hooking events

Instances of the hooking behavior that induces female spawning were counted by observing video play in superslow mode [9]. The video captured by the video camera (Panasonic HC-W580M) was converted to super slow mode by the video converting software Adobe Premiere Pro (Adobe Inc., San Jose, CA).

### Statistical analysis

All experiments were repeated more than three times. One-way analysis of variance (ANOVA) was performed using GraphPad Prism (San Diego, CA). A *P* value < 0.05 was considered statistically significant.

## Supporting information

S1 Fig Determination of half-life of compounds in water by HPLC analysis. (DOCX)

S1 Movie Representative chasing behavior by activated male fish. This movie is provided as a supplement to Fig 1.
(MP4)

S2 Movie Representative spawning behavior. This movie is provided as a supplement to Fig 1.
(MP4)

S3 Movie Representative hooking behavior in high-speed imaging analysis. This movie is provided as a supplement to Fig 1.
(MP4)

S1 Table Low data for Figs 2, 3 and 4. (XLSX)

S2 Table Low data of statistical analyses for Figs 2, 3 and 4. (XLSX)

S3 Table Incorporation of Tes into fish body during in vivo treatment. (XLSX)

S4 Table Relationship between Tes incorporation and fish body weight. (XLSX)
