# Supplementary material for: In vivo induction of male sexual behavior in zebrafish by adding agents to water
Source: PLoS One. 2024 Aug 1;19(8):e0300759. doi: 10.1371/journal.pone.0300759 (PMC11293745; doi:10.1371/journal.pone.0300759)
Supplement: S1 Fig — (DOCX) [file pone.0300759.s001.docx]

**S1 Fig** Determination of half-life of compounds in water by HPLC analysis.

A B


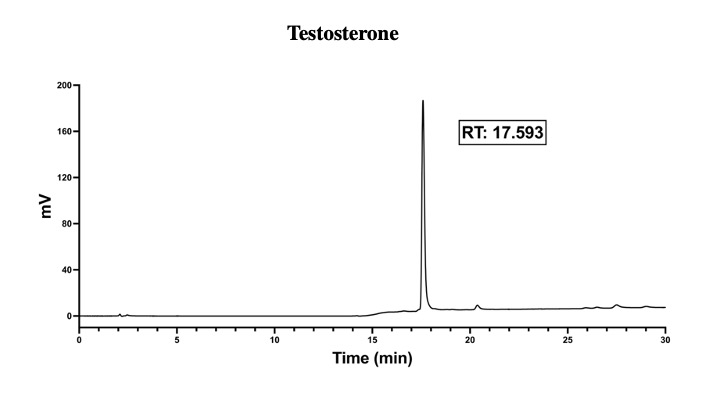
**
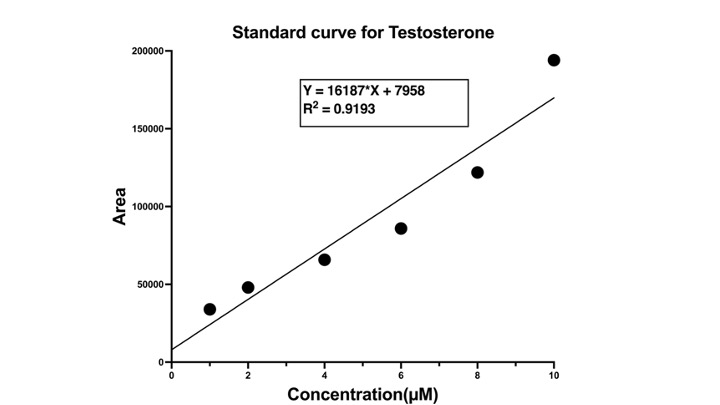
**

**C D**

**
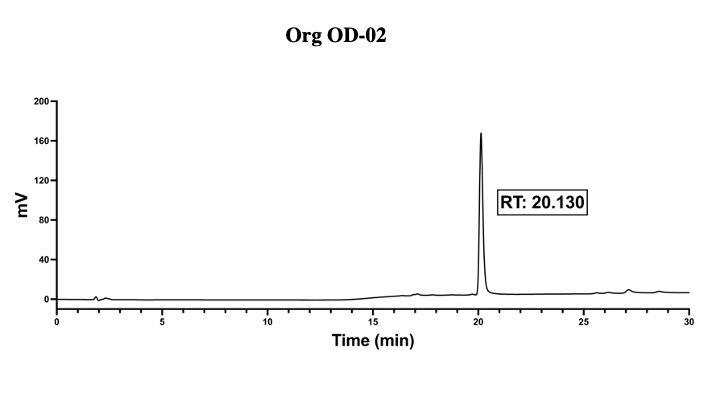

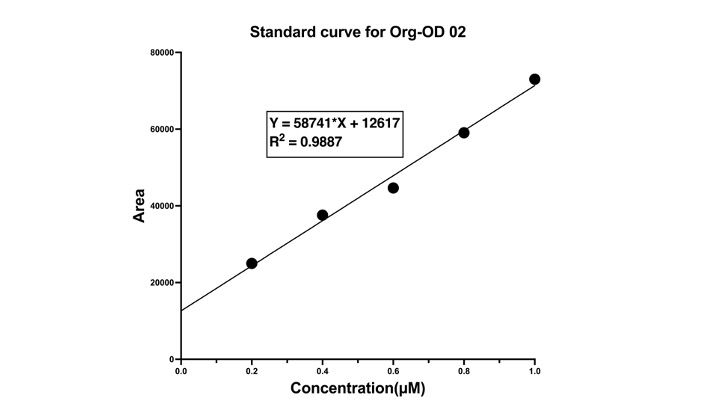
**

**E**

**
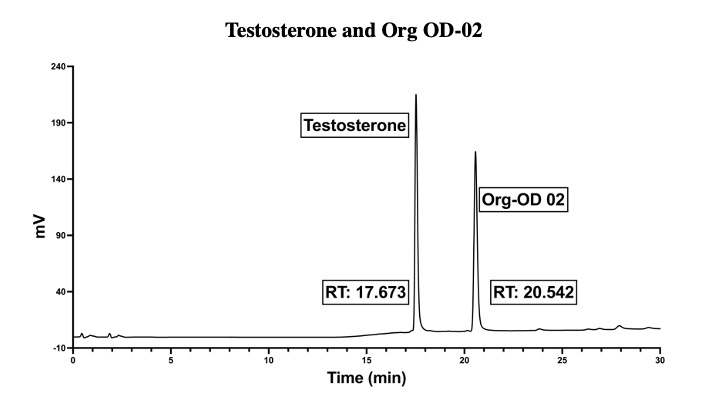
**

Representative chromatograms of Tes (A) and Org (C) on a C30-5 column are shown. The standard curve for the measurement of Tes (B) and Org (D) was constructed from the chromatogram of different concentrations of each compound. The peaks of Tes and Org were clearly separated (E), making it possible to measure the concentration in the mixture.

F G


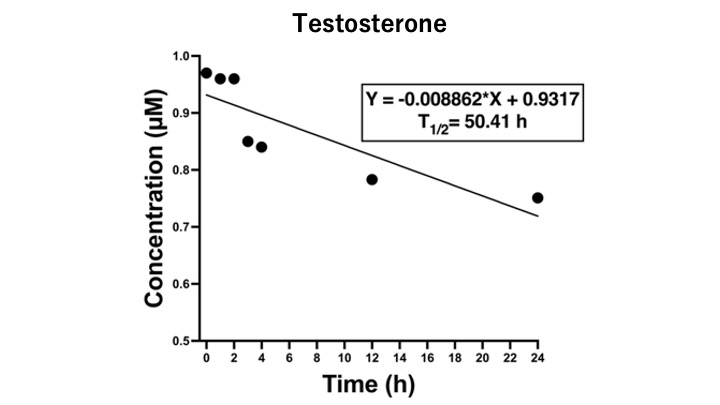

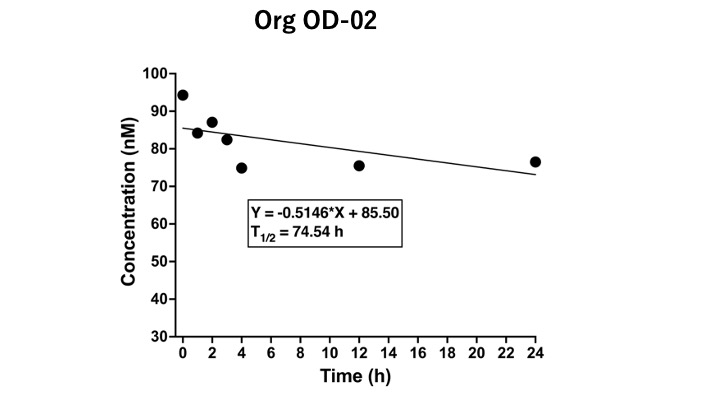


The half-life of Tes (F) and Org (G) in the in vivo experimental conditions was determined from the concentrations of each compound after treatment at different time points.

**HPLC analysis methods:**

Quantitative analysis of the prepared sample was performed by high-performance liquid chromatography (HPLC) methods. Wakopak Navi C30-5 column was used as the stationary phase (column size = 2.0*150mm), and a gradient mixture of acetonitrile and water with 0.05% trifluoroacetic acid (TFA) was used as the mobile phase. The column temperature was maintained at 40°C and the flow rate was 0.3 mL/min. Standard curves for testosterone and Org OD-02 were prepared using the peak area of known concentrations of chemicals. The unknown concentration of the chemicals was determined using the linear regression equation from the standard curve. In the incubated system water, the final concentration of Testosterone and Org OD-02 was 1 µM and 0.1 µM, respectively. 1 mL samples were taken from the incubated system water at different time points (0, 1, 2, 3, 4, 12 and 24 h). Each sample was injected three times for reliable data analysis.
